# Recent Advances in Electrochemiluminescence-Based Systems for Mammalian Cell Analysis

**DOI:** 10.3390/mi11050530

**Published:** 2020-05-22

**Authors:** Kaoru Hiramoto, Elena Villani, Tomoki Iwama, Keika Komatsu, Shinsuke Inagi, Kumi Y. Inoue, Yuji Nashimoto, Kosuke Ino, Hitoshi Shiku

**Affiliations:** 1 Graduate School of Environmental Studies, Tohoku University, Sendai 980-8579, Japan; kaoru.hiramoto.s5@dc.tohoku.ac.jp (K.H.); tomoki.iwama.s8@dc.tohoku.ac.jp (T.I.); keika.komatsu.p3@dc.tohoku.ac.jp (K.K.); kumi.inoue.b3@tohoku.ac.jp (K.Y.I.); 2 Department of Chemical Science and Engineering, School of Materials and Chemical Technology, Tokyo Institute of Technology, Yokohama 226-8502, Japan; villani.e.aa@m.titech.ac.jp (E.V.); inagi@cap.mac.titech.ac.jp (S.I.); 3 PRESTO, Japan Science and Technology Agency (JST), Kawaguchi 332-0012, Japan; 4 Graduate School of Engineering, Tohoku University, Sendai 980-8579, Japan; yuji.nashimoto.d8@tohoku.ac.jp; 5 Frontier Research Institute for Interdisciplinary Sciences, Tohoku University, Sendai 980-8578, Japan

**Keywords:** electrochemiluminescence (ECL), mammalian cell analysis, electrochemical device, electrochemical microscopy, single-cell analysis, ECL luminophore

## Abstract

Mammalian cell analysis is essential in the context of both fundamental studies and clinical applications. Among the various techniques available for cell analysis, electrochemiluminescence (ECL) has attracted significant attention due to its integration of both electrochemical and spectroscopic methods. In this review, we summarize recent advances in the ECL-based systems developed for mammalian cell analysis. The review begins with a summary of the developments in luminophores that opened the door to ECL applications for biological samples. Secondly, ECL-based imaging systems are introduced as an emerging technique to visualize single-cell morphologies and intracellular molecules. In the subsequent section, the ECL sensors developed in the past decade are summarized, the use of which made the highly sensitive detection of cell-derived molecules possible. Although ECL immunoassays are well developed in terms of commercial use, the sensing of biomolecules at a single-cell level remains a challenge. Emphasis is therefore placed on ECL sensors that directly detect cellular molecules from small portions of cells or even single cells. Finally, the development of bipolar electrode devices for ECL cell assays is introduced. To conclude, the direction of research in this field and its application prospects are described.

## 1. Introduction

Analysis using mammalian cells is essential in a wide range of areas, from fundamental studies in biology to modern medicine and clinical diagnosis. In the field of cellular biology, analysis at the single-cell level is essential to reveal cellular mechanisms due to the heterogeneity of individual cells, which cannot be seen in a large cell population. For the development of regenerative medicine, the fast diagnosis of cells is required to discriminate differentiation and canceration. Moreover, recent progress in precision medicine relies on cell-based assays using samples from real patients for the screening of drug effects. In addition, the transplantation of cultured cells is of particular interest in the context of regenerative medicine. Modern cellular analysis is therefore required to achieve not only a high sensitivity and selectivity, but also a real-time, high-throughput, and comprehensive detection.

Electrochemiluminescence (ECL) is an analytical technique that utilizes electrochemical potentials to produce photoluminescence, and several reviews of ECL as an analytical tool have been published to date. Owing to its integration of electrochemical and spectroscopic methods, ECL exhibits a number of advantages, including a high sensitivity, low background signal, high spatial resolution, high throughput, and simple instrumentation setups [1,2]. Furthermore, the possibility of controlling the light emission both temporally and spatially through the application of a suitable potential has fostered the development of imaging techniques based on ECL [3,4]. In addition, since the cell-based assay has become increasingly important in biological and clinical fields, ECL analysis has gathered significant attention in these fields due to its versatile and remarkable features. Indeed, tremendous research efforts have been made in this area in the past decade. Therefore, this review focuses on recent developments in ECL techniques, in particular in the context of their application in mammalian cell analysis.

**Figure 1:**
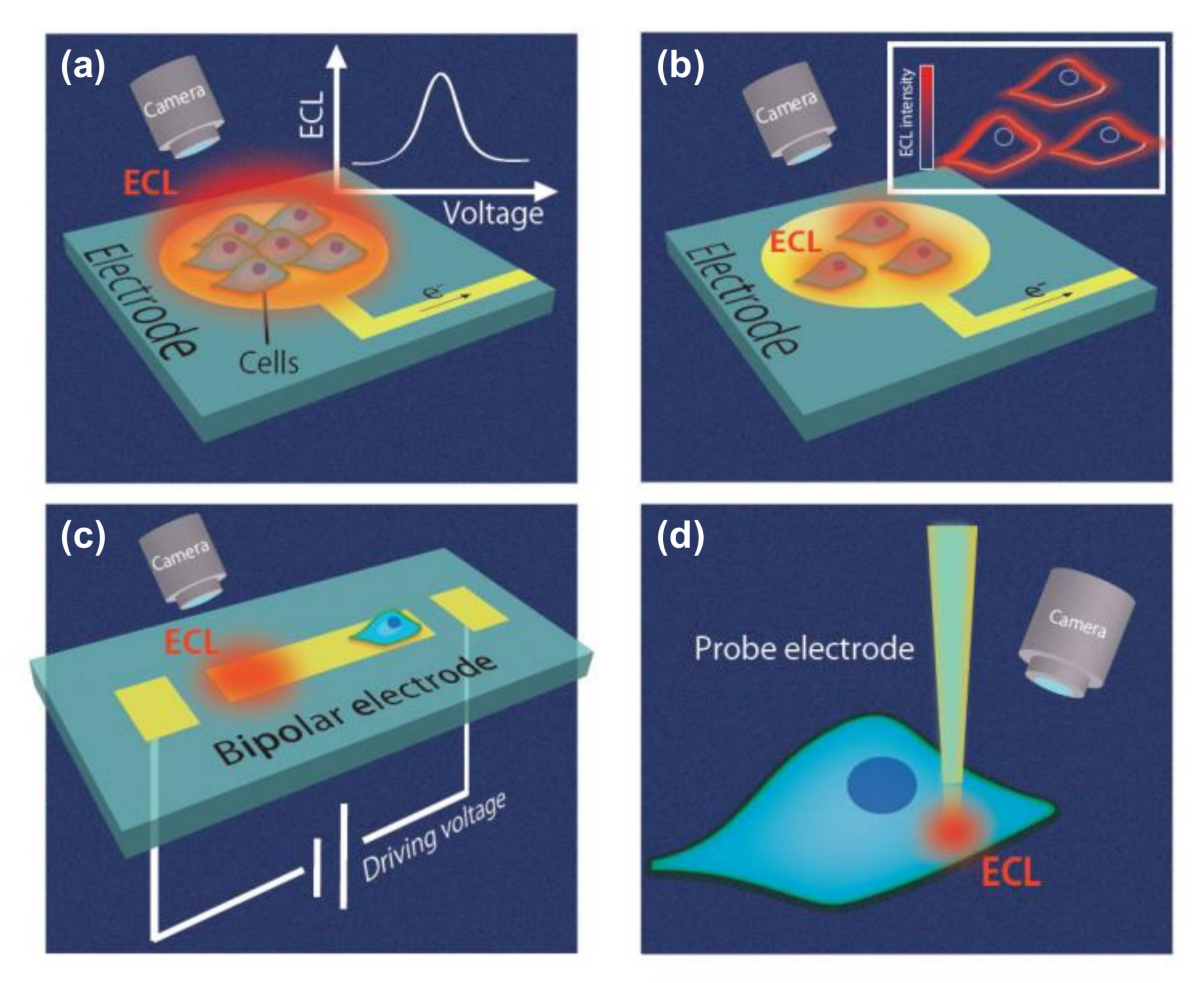
Various electrochemiluminescence (ECL) devices for cell analysis. (**a**) Chip devices not for microscopic imaging. (**b**) ECL microscopes. (**c**) Bipolar electrode (BPE) devices. (**d**) Probe devices.

The key components of an ECL system are the luminophores used as signal probes and the electrode devices that induce the chemical reactions of the luminophores. Various types of ECL electrode devices exist, including chip (Figure 1a–c) and probe devices (Figure 1d). In chip devices, an electrode is set and cells or cellular extracts are introduced. Subsequently, ECL signals are obtained (Figure 1a); as a result, these chip devices are useful for simple analysis. For ECL microscopy (Figure 1b), the ECL signals are obtained using a microscope, and target analytes can be visualized at the single-cell level. Another chip device, the bipolar electrode (BPE), is also widely used for ECL analysis (Figure 1c) due to its ability to function wirelessly, as discussed later. Such chip devices are useful for high-throughput analysis. In terms of intracellular analysis, probe devices have been proposed (Figure 1d), and these probe devices can then be combined with BPE systems. Cell analysis using these devices is described in later sections, and these devices are summarized in a later table.

In the second section of this review, an ECL light-emitting process is described by highlighting two luminophores, namely ruthenium and luminol derivatives, which are mainly used in cellular analysis. Although the method of ECL was first reported in the early 1960s, the most significant progress in terms of ECL for bioanalytical applications occurred upon the development of an emitting process in an aqueous medium. Among the various luminophores used in ECL detection, ruthenium complexes and luminol derivatives are commonly utilized for cellular analysis due to their high solubility in aqueous media. Indeed, ruthenium and luminol are now the most common luminophores that support the fundamental ECL reaction in biological samples. The various types of ECL systems available for cell analysis are summarized in a later table.

The third section of this review introduces imaging techniques that employ the ECL system. Although several methods based on fluorescence microscopy have been proposed for single cell analysis [5], ECL is also receiving significant attention in the context of single-cell imaging, owing to its high spatial and temporal resolutions.

Subsequently, the fourth section focuses on ECL sensors. In the past decade, ECL sensors have been successfully developed for use in commercial immunoassays involving complex physiological samples, such as blood and urine. However, the in situ sensing of cell-derived molecules in a small portion of cells or at the single-cell level is still in development. In the first subsection, we introduce ECL platforms for detecting intracellular molecules such as glucose, reactive oxygen species (ROS), and glutathione in a single cell. We then focus on sensing strategies and systems for the detection of specific cells by capturing proteins expressed on cell surfaces or the DNA and small molecules derived from cells. In this field, ECL is often combined with nanotechnology, and so this section also covers ECL systems incorporating quantum dots, metal- and carbon-based nanomaterials, and their combinations.

In the fifth section, we summarize a BPE system for the ECL analysis of cells. A BPE is a conducting material in an electrolytic solution to which a potential is applied between two driving electrodes (Figure 1c). When the potential is sufficient, anodic and cathodic reactions are induced at both ends of the BPE. By monitoring the ECL at one of the poles, the electrochemical reaction at another pole can be evaluated because the anodic and cathodic currents at the BPE are equal and the ECL emission depends on the currents. Since BPEs can be controlled wirelessly with only a single power supply, a large number of electrochemical sensors can be easily incorporated without complicated wiring. BPEs can be used not only for bioanalysis but also for polymer synthesis [6,7] and hydrogel electrodeposition [8]. However, the present review focuses on mammalian cell analysis using BPE–ECL systems.

Since many types of ECL systems are introduced in this study, we summarize the ECL systems in tables based on their types of electrode devices, target analytes, and emitting species. Finally, we discuss the direction of research in this field and its application prospects.

Recently, excellent reviews regarding ECL have been published, including a minireview on the ECL single-cell analysis that has emerged in recent years [9] and a review on pathogenic bacteria detection [10]. In contrast, in this present review, recent studies ranging from single-cell systems to cell aggregates and cell secretions, focusing on mammalian cells, are reviewed to give a comprehensive summary.

## 2. Luminophores for Cellular Analysis

The choice of emitting species in ECL comprises a wide variety of systems ranging from organic to inorganic compounds [11,12], carbon nanomaterials [13], quantum dots [14], nanoparticles [15], and their assemblies. Nevertheless, ruthenium complexes, luminol, and their derivatives are the most employed luminophores for cell analysis due to their high luminescence efficiency, solubility in an aqueous medium, and the ability to achieve efficient ECL emission at a physiological pH.

In the following subsections, we review the development of the above-mentioned luminescent species, their mechanistic pathways of light generation, and their application in ECL cellular analysis.

### 2.1. Ruthenium Based Systems

Tris(bipyridine)ruthenium(II), Ru(bpy)_3_^2+^ was the first inorganic complex reported to exhibit ECL properties [16]; indeed, it still represents the benchmark system in both fundamental studies and commercial applications. Its success is due to a number of reasons, including its solubility and strong luminescence in both organic and aqueous solvents at room temperature and its ability to undergo reversible one-electron transfer reactions at easily accessible potentials, thereby leading to stable reduced and oxidized species.

ECL generation from the Ru(bpy)_3_^2+^ complex was initially achieved in acetonitrile via the annihilation mechanism [16], in which both the radical cation and the radical anion of the complex were concomitantly generated at the electrode surface after switching the potential to oxidation and reduction steps, respectively. Successively, the ECL of Ru(bpy)_3_^2+^ using coreactant species such as oxalate [17] and peroxydisulfate [18] was reported. However, the turning point in the history of ECL was the employment of tri-*n*-propylamine (TPrA) as a coreactant [19], which allows efficient ECL emission in aqueous media in addition to at a physiological pH (~7.4). Both of these characteristics are of paramount importance for practical applications in cellular analysis.

**Scheme 1:**
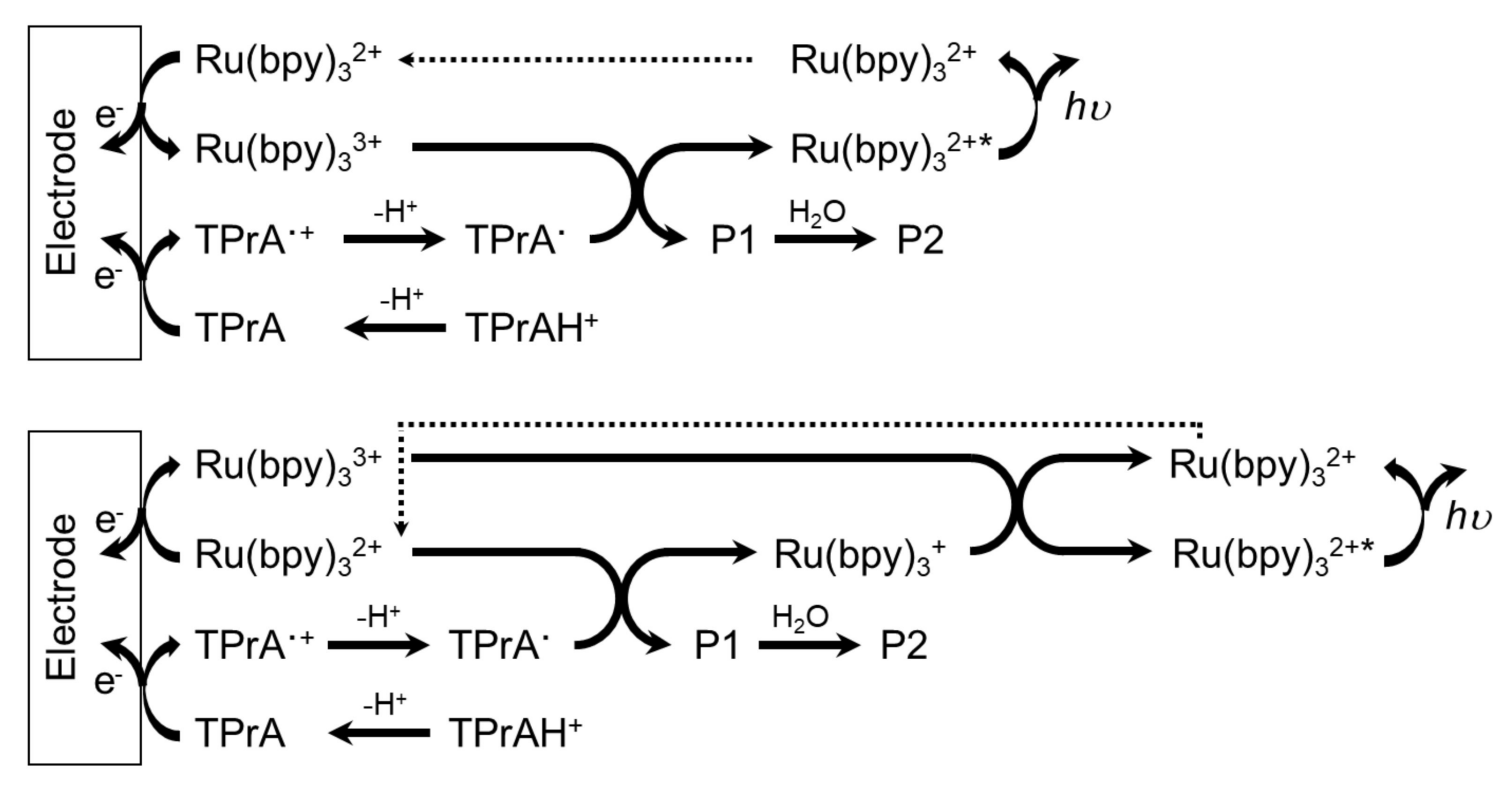
The two ECL routes of the Ru(bpy)_3_^2+^-tri-*n*-propylamine (TPrA) system, in which Ru(bpy)_3_^2+^ and TPrA are both oxidized directly on the electrode.

ECL generation from the Ru(bpy)_3_^2+^/TPrA system involves several mechanistic pathways depending on the precursor concentrations and whether Ru(bpy)_3_^2+^ is free to diffuse in solution or anchored to a solid phase [20] such as beads or nanoparticles. However, TPrA undergoes an irreversible electrochemical oxidation, generating the TPrA radical cation (TPrA^•+^), which, after deprotonation, leads to the formation of the TPrA radical (TPrA^•^), a strong reducing agent. Both radicals are involved in the generation of the Ru(bpy)_3_^2+^ excited state, which then relaxes to the ground state, emitting a photon at ~620 nm (orange emission) and regenerating the luminophore at the end of the process. When the Ru(bpy)_3_^2+^ luminophore is free to diffuse in solution, the luminophore and coreactant are involved in electrochemical oxidation directly at the electrode surface, as described by the two main routes in Scheme 1 [20]. In the context of cell imaging, the free diffusion of Ru(bpy)_3_^2+^ and *N*-(2-hydroxyethyl)piperazine-*N*’-2-ethanesulfonic acid (HEPES) coreactant was also employed [21]. In addition, the Ru(bpy)_3_^2+^/glutathione (GSH) system has been reported for cell analysis [22].

**Scheme 2:**
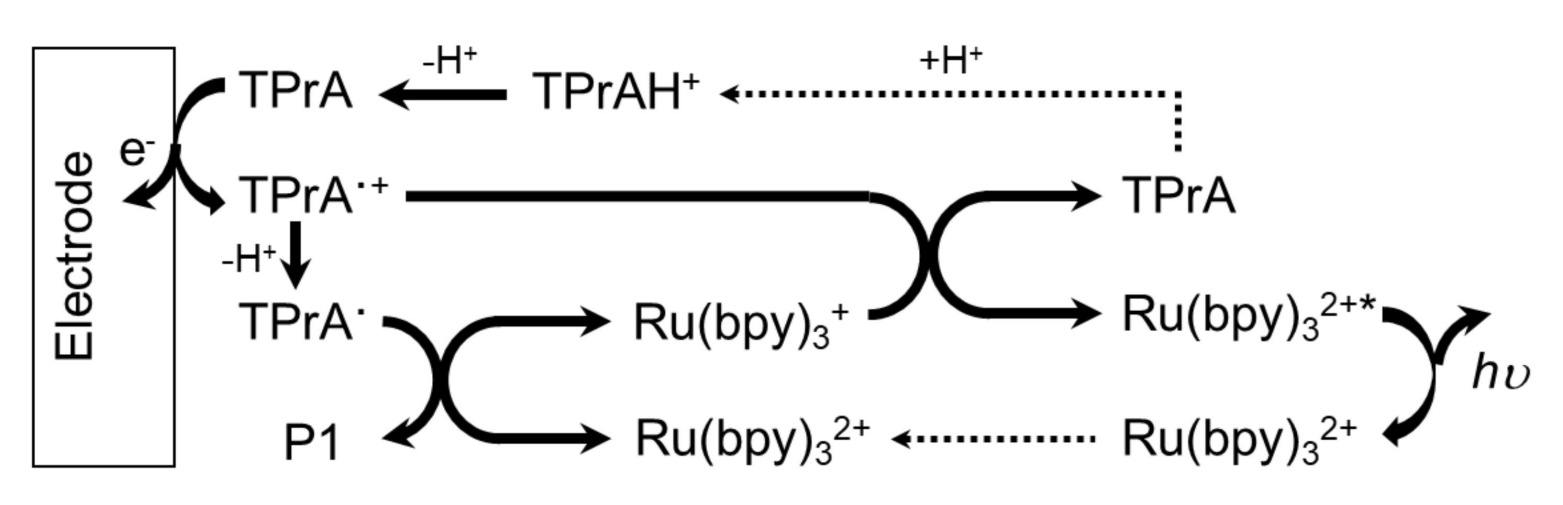
The possible ECL route of the Ru(bpy)_3_^2+^-TPrA system in which Ru(bpy)_3_^2+^ is not directly oxidized on the electrode but reacts with electrogenerated TPrA radicals.

However, another strategy often encountered in cell studies concerns the immobilization of the Ru(bpy)_3_^2+^ complex as an electrochemiluminescent tag on a solid phase, such as nanoparticles, beads, or directly on cell membranes (see Section 4.2 for further details). In such a case, the direct oxidation of the luminophore at the electrode surface is hindered, and the formation of its excited state is realized by the electrogenerated radicals of the coreactant (Scheme 2 [20]). For example, the antibody–antigen interaction can be exploited to anchor the Ru(bpy)_3_^2+^ complex on cell membranes. In this case, one of the bipyridine moieties of the Ru(bpy)_3_^2+^ complex is conveniently functionalized with the *N*-succinimidyl ester (Ru(bpy)_2_-bpy-CO-OSu), and such a modified complex can be successively linked on an antibody.

The feasibility of using nanoparticles in analytical applications has gained increasing attention in the ECL community [15], since they represent an ideal nanocomposite matrix for the immobilization of a large number of Ru(bpy)_3_^2+^ luminescent tags to promote highly efficient ECL emission. For instance, Ru(bpy)_3_^2+^ probes can be covalently attached to the surface of silica nanoparticles or loaded inside the nanoparticles, or a combination of both strategies can be exploited to achieve extremely high ECL intensities for improved sensitivity [23]. Moreover, gold nanoparticles [24,25,26], quantum dots [27], and magnetic beads [28,29] have been employed to decorate the nanoarchitectures and further improve the ECL emission.

### 2.2. Luminol-Based Systems

Luminol (5-amino-2,3-dihydro-1,4-phthalazinedione) is another organic system commonly used for ECL cellular analysis. The first report regarding light generation from an aqueous alkaline solution of luminol at an electrode surface appeared in 1929 [30].

**Scheme 3:**
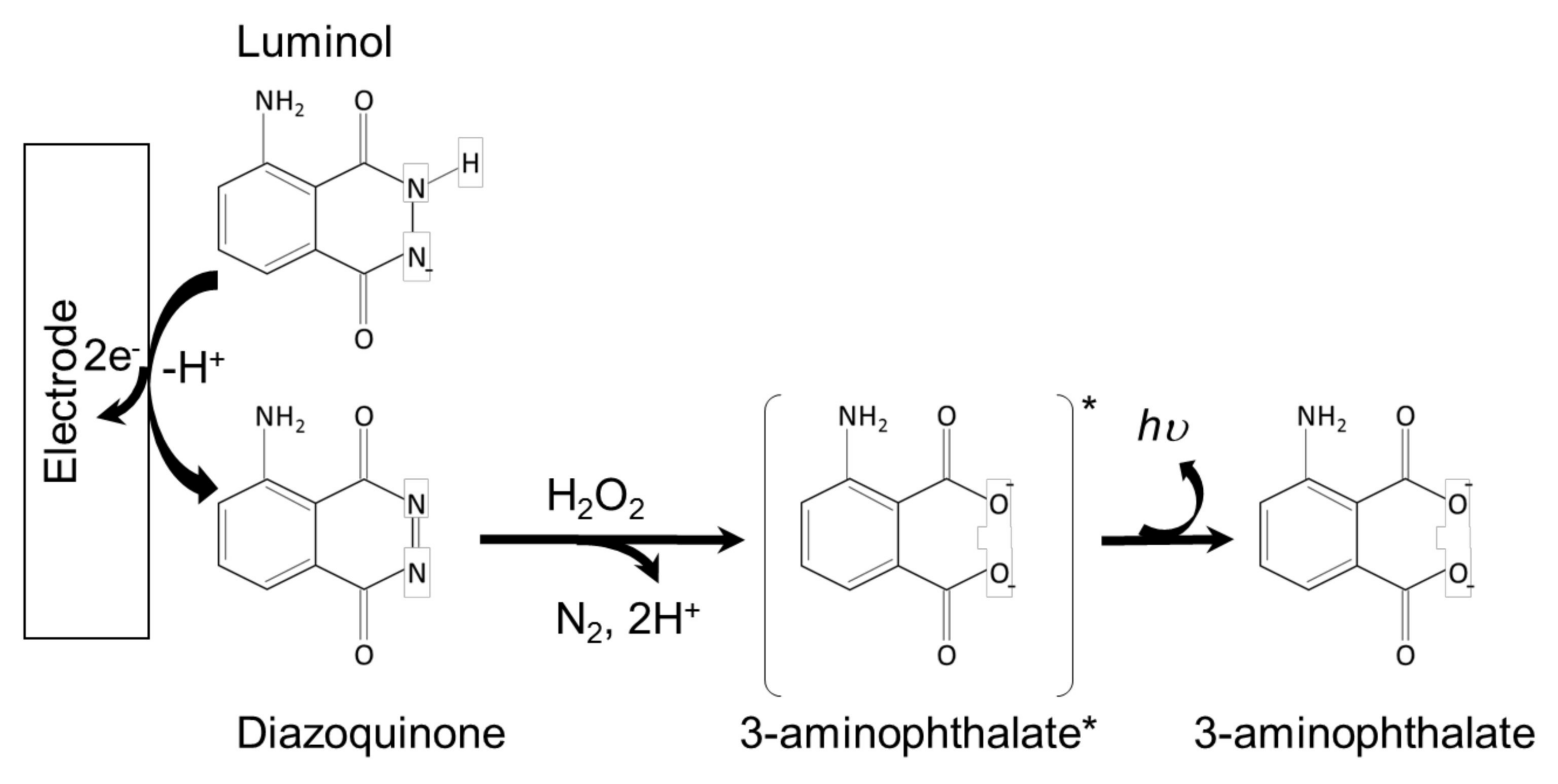
The ECL mechanism of the luminol-H_2_O_2_ system.

Several mechanistic pathways have been proposed for luminol ECL generation depending on the applied potential and experimental conditions [31]. Generally, luminol is deprotonated in aqueous solutions to generate an anion that can undergo electrochemical oxidation to a diazaquinone form. In the presence of hydrogen peroxide, this intermediate species undergoes further oxidation to 3-aminophtalate in its excited state, which subsequently relaxes to the ground state through the emission of a photon in the typical blue range (*λ*_ECL_ = 425 nm) (Scheme 3 [32]).

Hydrogen peroxide is generally an integral part of the majority of luminol ECL-related studies. Since hydrogen peroxide is generated in several biological processes or released from cells through oxidative stress, its detection is usually coupled with luminol ECL (see Section 3.2 for further details). For instance, hydrogen peroxide is produced by the oxidase enzymes so that the corresponding enzymatic substrates such as glucose and cholesterol can be detected. Other reactive oxygen species (ROSs), such as the peroxide anion HOO^−^ and the superoxide radical O_2_^•−^, can accelerate the generation of the 3-aminophtalate excited state to enhance the ECL emission of luminol.

In contrast to Ru(bpy)_3_^2+^, which is regenerated at the end of the ECL process, luminol is irreversibly oxidized to a nonrenewable species. This factor, together with the strong basic conditions required to generate a sufficient ECL emission and the high nonspecific background signal—likely due to the generation of oxygen in aqueous solution at an anodic potential—may limit the potential application of these systems in cellular analysis. Nevertheless, the luminol ECL system requires a lower anodic potential than Ru(bpy)_3_^2+^ on an available electrode such as indium tin oxide (ITO) (i.e., approximately +0.4 V vs +1.2 V for the ruthenium complex, with Ag/AgCl as the reference electrode), which is a favorable factor for the imaging studies of living cells. Furthermore, the low oxidation potential is beneficial in terms of electrode fouling resistance and long-term stability, thereby rendering luminol a more compatible system for a large variety of working electrode materials compared to Ru(bpy)_3_^2+^. Generally, a compromise must be found between the choice of system and the desired study to be performed.

A luminol analogue, dedicated to the analysis and imaging of cellular systems in neutral solutions, is the 8-amino-5-chloro-2,3-dihydro-7-phenyl-pyrido(3,4-d)pyridazine-1,4-dione sodium salt, which is simply referred to as L012 sodium salt. Analogous to its luminol precursor, the L012 anion can be electrochemically oxidized but it generates a stronger luminescence. For this reason, it is widely employed for cell imaging and the detection of species of relevant biological interest, such as glucose and cholesterol.

*N*-(4-aminobutyl)-*N*-ethylisoluminol (ABEI) is another luminol analog that has found applications in the field of cell analysis. It exhibits ECL emission when oxidized in basic aqueous solutions, and the addition of hydrogen peroxide improves its emission efficiency. It has been shown that ABEI can be easily incorporated in the skeleton of porous conducting polymer hydrogels, providing a short diffusion distance between the luminophore and the hydrogen peroxide coreactant, thereby realizing a sensitive in situ monitoring of the oxygen radicals released from cells [33]. It has also found application as a highly efficient ECL signal probe for cancer cell analysis [34].

## 3. Electrochemiluminescence (ECL) Microscopy for Cell Imaging

Conventional electrochemical imaging has been used for bioanalysis, whereby local electrochemical reactions are monitored using electrochemical devices/systems. For example, scanning electrochemical microscopy (SECM) is widely utilized, and is an excellent tool that can perform chemical mapping at the nanometer scale [35]. However, SECM requires a skilled operator, and the collection of the electrochemical image is slow due to probe scanning. Electrode arrays containing many individual electrochemical sensors have therefore been proposed for the electrochemical imaging of cells without scanning a probe [36,37]. In addition, complementary metal-oxide semiconductor (CMOS)-based electrode array devices have recently been proposed to perform the real-time electrochemical imaging of biosamples [38]. However, the number of electrochemical sensors is limited and the resolution is low. Thus, to solve these issues, ECL can be employed, since the electrochemical reactions can be converted to ECL signals and the signals can be measured using an optical microscope. Basically, the spatial resolution of ECL imaging is relevant to the diffusion limit of luminophores and coreactants. In addition, owing to the relatively short life time of the radicals involved in the ECL system (i.e., TPrA^•^ and TPrA^•+^ in the case of Ru ECL), the strong emission of ECL occurs in the vicinity of the electrode surface, and the detection of intracellular molecules is possible using such surface-confined microscopy or lightning probes. Thus, in this section, we introduce recent reports regarding single cell imaging, cell adhesions, membrane proteins, and released cellular molecules. Such ECL microscopy techniques for single-cell analysis can be considered powerful tools for application in modern medicine and biology.

### 3.1. Imaging of Cell Membranes and Cell Adhesion

**Figure 2:**
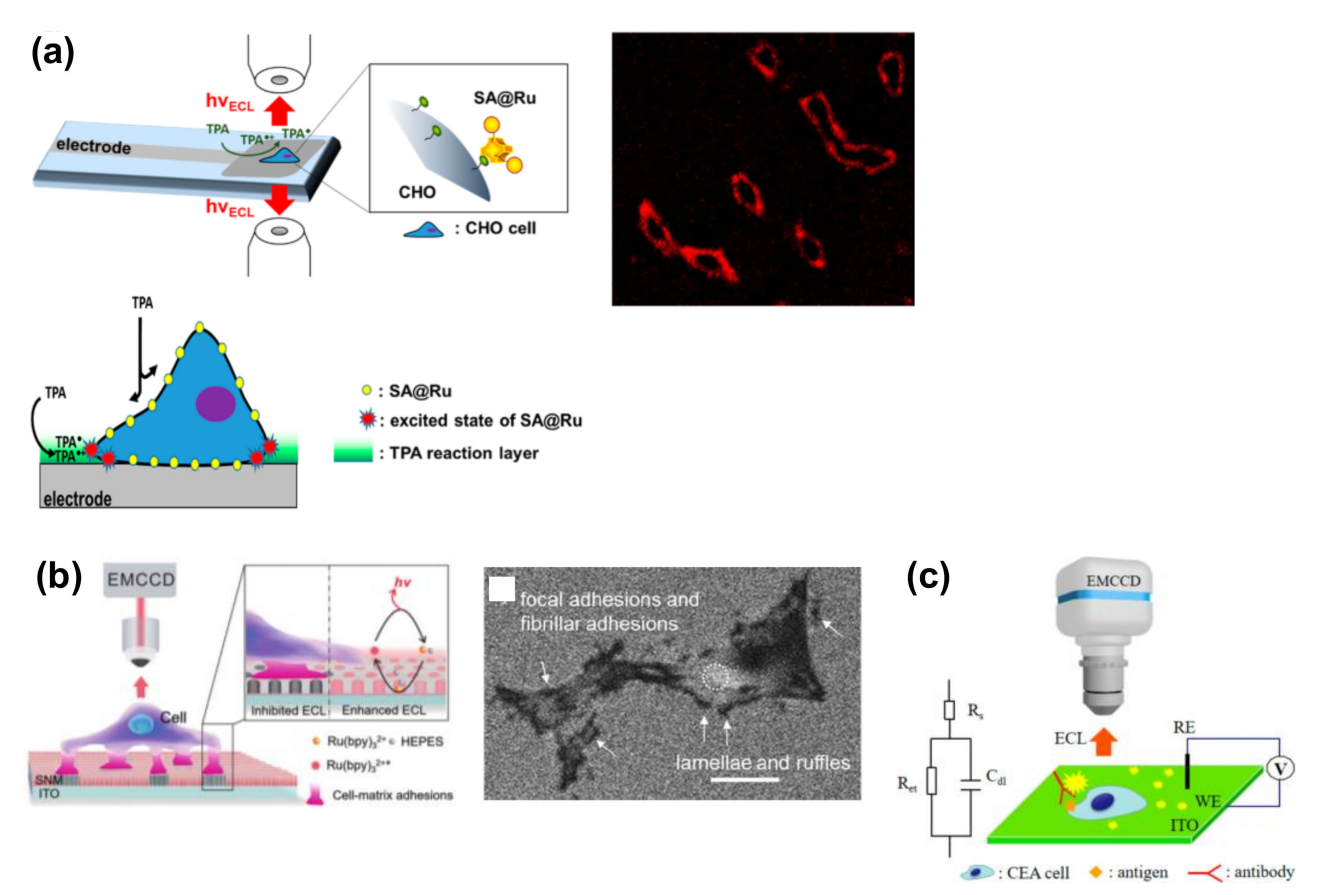
ECL imaging of cell membranes and cell adhesion. (**a**) ECL imaging of cell membranes. Chinese hamster ovary (CHO) cells were modified with biotin, and streptavidin-modified Ru(bpy)_3_^2+^ (SA@Ru) was then attached to the membranes. Reproduced with permission from [40]. (**b**) ECL imaging of cell-matrix adhesions. PC12 cells were cultured on the electrode. Scale bar = 20 µm. Reproduced with permission from [21]. (**c**) ECL-based capacitance microscopy for the imaging of antigens on cells. Carcinoembryonic antigen (CEA) was labeled with an anti-CEA antibody. Reproduced with permission from [41].

Figure 2 shows a summary of the ECL imaging of cell membranes and cell adhesion. In this context, Sojic and coworkers reported the ECL imaging of the cellular membranes of single cells [39,40] using Ru(bpy)_3_^2+^ as a label for cellular membranes. In this case, as Ru(bpy)_3_^2+^ was modified on the cell membrane, the ECL emission was triggered only by the electrochemical oxidation of TPrA (Scheme 3). Since TPrA is not permeable to the cell membrane, only those diffused between the cell membrane and the electrode surface could contribute to the luminescence, resulting in a surface-confined image of basal cell membranes (Figure 2a). During detection, a potential of 1.3–1.4 V was applied to a glassy carbon electrode in a TPrA solution, and this method was named surface-confined ECL microscopy. It allowed the visualization of not only cellular membranes, but also the epidermal growth factor receptor (EGFR). During detection, the EGFR of MCF10A was modified using antibodies labeled with Ru(bpy)_3_^2+^ (Ab@Ru) [39]. Only the basal membrane of the cell became luminescent, thereby indicating that this method reveals details that are not resolved by classic fluorescence microscopy.

ECL microscopy was also applied for visualizing apoptosis at a single-cell level via the detection of the cellular membrane components, i.e., the EGFR and phosphatidylserine (PS) [42]. During detection, the target cells were modified with ECL tags, namely Au@L012 and g-C_3_N_4_ functionalized with EGF and a peptide to recognize PS. Both L012 (luminol analog) and g-C_3_N_4_ generate strong ECL in the presence of the coreactant H_2_O_2_ at different electrochemical potentials. Cells with the ECL tags were modified on the carbon nanotube sponge as an electrode, and the corresponding expressions were visualized simultaneously at different potentials.

In addition, Su and coworkers reported an ECL microscopic method for the label-free imaging of the cell-matrix adhesions of single living cells (Figure 2b) [21]. For this detection, an indium tin oxide (ITO) electrode was modified with a silica nanochannel membrane (SNM). Target cells were cultured on the electrode to form cell-matrix adhesions, and ECL generation involving Ru(bpy)_3_^2+^ was stimulated by applying a potential of +1.3 V in HEPES solution, which served not only as a “Good” buffer but also as a coreactant of Ru(bpy)_3_^2+^. Since the adhesions formed close contacts with the electrode surface, ECL reactions were inhibited. Therefore, under ECL microscopy conditions, dark images were obtained for the cells. Thanks to the negatively charged SNM, which could concentrate the oppositely charged Ru(bpy)_3_^2+^, a strong luminescence was observed at the non-contact site of the cell membrane. Moreover, the study emphasized that the vertically aligned channels of SNM allow the limited diffusion of luminophores, whereby the cellular domain of the adhesion site and non-adhesion site could be distinguished. Although the lamellae and ruffles along the entire cell periphery were too thin to be observed in classic bright field images, these structures were clearly visualized by the ECL system without any labeling.

ECL-based capacitance microscopy using a square-wave voltage was developed to visualize membrane antigens using an antibody (Figure 2c) [41]. ECL signals depend on the capacitance at the electrode; in this context, it is important to note that when a nonconductive biospecies is bound at the electrode surface, the capacitance decreases. This decreased capacitance is proposed to result in a relatively high potential drop across the double layer in this region, which in turn induces an increase in the luminol-based ECL intensity. During detection, a square-wave voltage of high frequency was applied and the frequency was also found to affect the potential drop. Moreover, at suitable frequencies, carcinoembryonic antigen (CEA) was successfully visualized in the ECL images. Since this antibody was used without the requirement for any ECL tags, this strategy can be considered superior to conventional methods.

### 3.2. Imaging of Released Cellular Molecules

**Figure 3:**
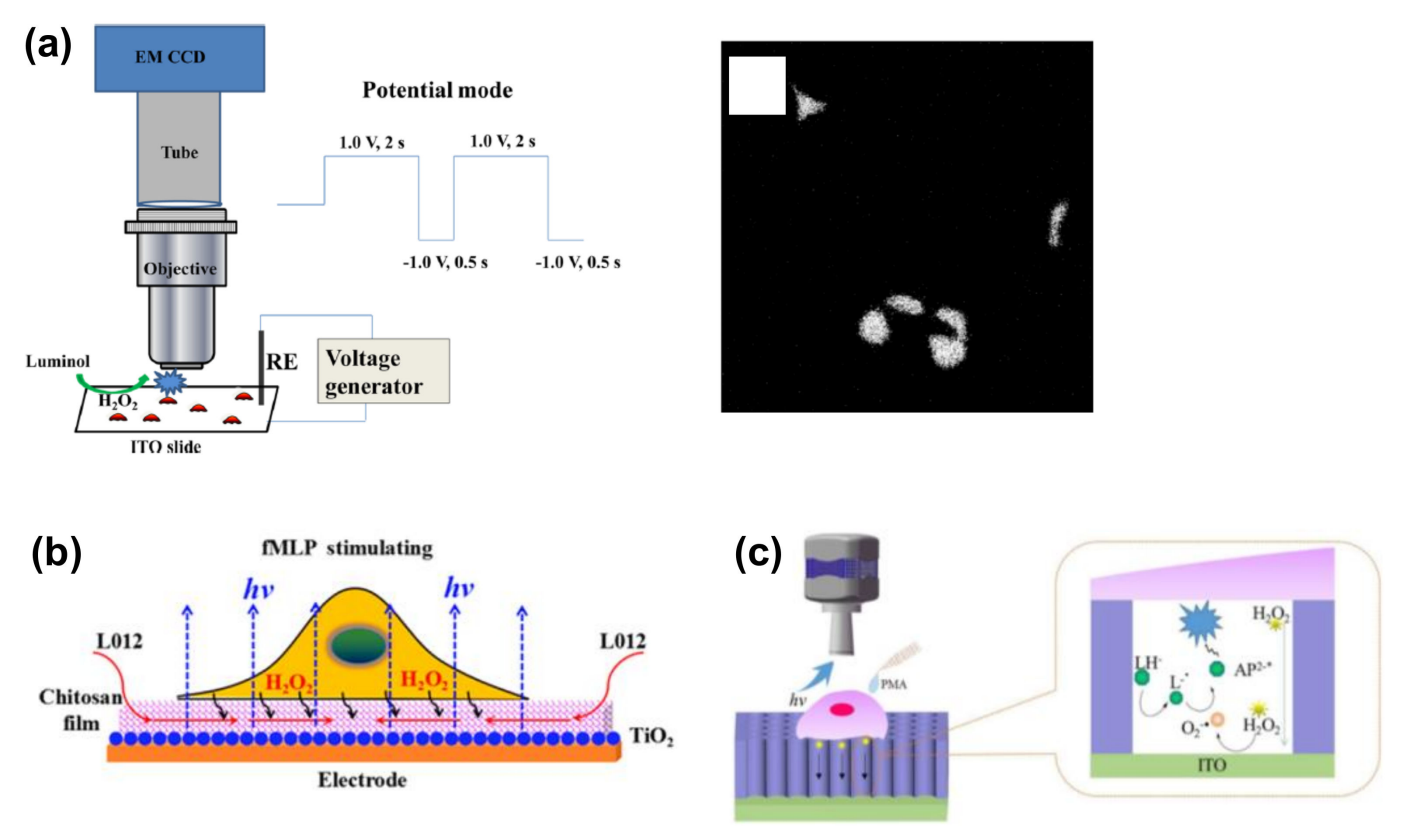
ECL imaging of H_2_O_2_ released from single cells. (**a**) A double-potential mode was demonstrated to obtain differential ECL images of H_2_O_2_ before and after stimulation by phorbol myristate acetate (PMA). Reproduced with permission from [43]. (**b**) The chitosan film was modified on an electrode to improve the diffusion of ECL chemicals. *N*-Formylmethionyl-leucyl-phenylalanine (fMLP) was used for the stimulation. Reproduced with permission from [44]. (**c**) Silica mesochannels (SMCs) were modified to improve the sensitivity. LH^−^: L012 anion; AP^2−^: 3-aminophthalate anion. Reproduced with permission from [45].

ECL imaging based on luminol analogues was also applied for the visualization of released cellular molecules such as H_2_O_2_ (Figure 3) [43,44,45], which are involved in various cellular functions, including signal transduction pathways. For example, to reduce electrode fouling due to positive potential, the potential was switched between 1.0 and −1.0 V in one study (Figure 3a) [43]. In addition, HeLa cells were cultured on an ITO electrode and stimulated with phorbol myristate acetate (PMA) for the induction of H_2_O_2_ efflux; the evolved H_2_O_2_ was successfully visualized in the ECL image at the single-cell level. Since H_2_O_2_ is a product formed upon the reaction between a substrate and an enzyme, the reaction can be visualized via H_2_O_2_ detection. Furthermore, the same group also reported the ECL imaging of active membrane cholesterol via H_2_O_2_ detection coupled with the reaction between cholesterol and cholesterol oxidase. Although ECL microscopy was not applied, other reports showed the ECL analysis of cholesterol for single-cell analysis using a pinhole [46,47], in addition to the co-determination of the active and inactive membrane cholesterol [48]. ECL imaging was also applied for the analysis of total cholesterol at the single-cell level [49].

Although ECL microscopy is a powerful tool due to the presence of zero cellular background light, electrode insulation is challenging due to cell adhesion. To address this issue, chitosan film can be coated on the electrode to prepare a space between the cells and the electrode, whereby luminol was found to diffuse easily through this space (Figure 3b) [44]. Moreover, to improve the spatial resolution of ECL imaging, vertically ordered silica mesochannels (SMCs) were prepared on ITO electrodes (Figure 3c) [45].

ECL microscopy was also applied for the detection of single semiconductive titanium dioxide (TiO_2_) nanoparticles for the visualization of H_2_O_2_ efflux from single cells [50]. During detection, the cells were cultured on ITO slides modified with semiconductive TiO_2_ nanoparticles and stimulated with PMA to release H_2_O_2_. The released H_2_O_2_ reacted with the TiO_2_ nanoparticles under the applied voltage, thereby inducing a high ECL. Through the use of various oxidases, several kinds of cellular molecules can therefore be monitored.

## 4. ECL Sensors for Cell Analysis

Due to the high heterogeneity of cells, single-cell analysis has become of great importance for cellular analysis in both fundamental and clinical research. Since the amounts of biomolecules and specific antigens in single cells are extremely small, highly sensitive detection is desired. In this section, we introduce ECL sensors and their associated techniques to achieve ultra-trace detection for the identification of cellular functions of small portions of cells or even single cells.

### 4.1. ECL Detection of Intracellular Molecules

**Figure 4:**
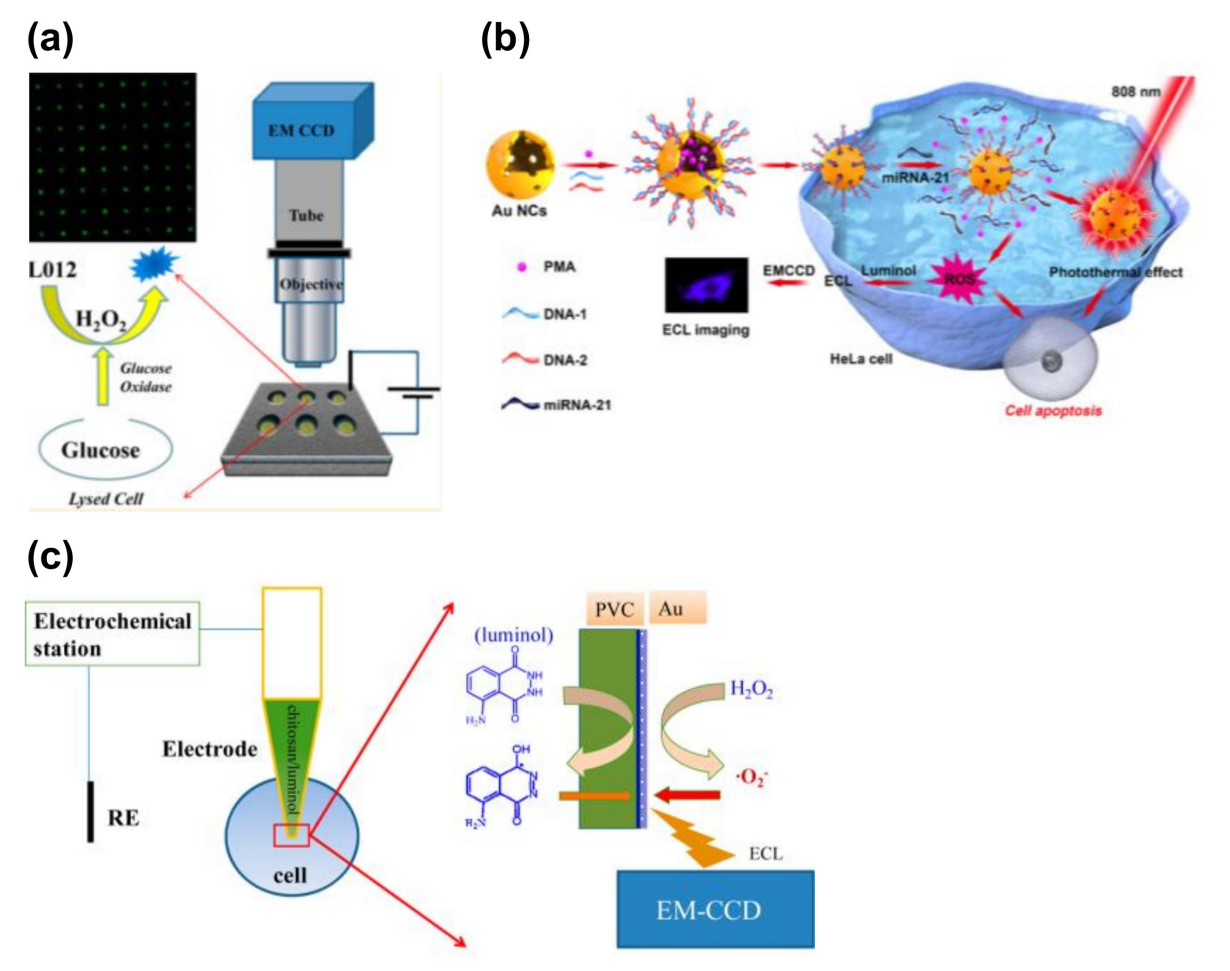
ECL detection of intracellular molecules. (**a**) ECL detection of intracellular glucose. Individual cells were trapped inside the microwells with an indium tin oxide (ITO) electrode. Cells were lysed to release glucose, and the glucose was reacted with glucose oxidase to produce H_2_O_2_. The H_2_O_2_ was visualized using ECL imaging. Reproduced with permission from [52]. (**b**) ECL detection of intracellular microRNA (miRNA). Au nanoclusters (NCs) were introduced as a probe, and reactive oxygen species (ROS) were released after the miRNA recognition. The ROS were visualized using luminol at the fluorine-doped tin oxide (FTO) electrode. Reproduced with permission from [55]. (**c**) ECL detection of intracellular H_2_O_2_ using a capillary electrode. Chitosan/luminol was filled with the tip. Intracellular H_2_O_2_ was converted into the ECL signals. Reproduced with permission from [56].

In this section, we summarize the ECL detection of intracellular molecules (Figure 4). Indeed, a number of electrochemical approaches have been applied for intracellular analysis, and these techniques have been summarized in a previous review [51]. For example, Xu et al. utilized the luminol−hydrogen peroxide system for the detection of intracellular glucose in single cells [52]. To achieve a rapid and high-throughput single-cell analysis, individual cells were trapped in microwell arrays on a gold-coated ITO slide. The cells were treated with luminol, Triton X-100, and glucose oxidase simultaneously. Upon treatment with Triton X-100, intracellular glucose was released into the microwells. This glucose was reacted with glucose oxidase to produce H_2_O_2_, which induced the luminol ECL. Using this strategy, intracellular glucose was successfully visualized in the ECL images. In addition, the same group reported a gold-coated polydimethylsiloxane (PDMS) chip with cell-sized microwells to improve the luminescence and simplify the device fabrication process (Figure 4a) [53].

The Zhang group reported the detection of intracellular telomerases present in cancer cells. More specifically, polyluminol–platinum nanoparticle (Pt NP) composite films modified with aptamer DNA were prepared on an ITO electrode as the ECL substrate to ultimately capture HL-60 cancer cells. Mesoporous silica nanoparticles (MSNs) filled with phorbol 12-myristate 13-acetate (PMA) were sealed by the telomerase primer (T-primer) DNA and the aptamer. Following the capture of HL-60 cells, the T-primer DNA on MSN@PMA could be moved away from the MSN@PMA surface after extension by telomerase in the HL-60 cancer cells. The released PMA stimulated the production of ROS in the cancer cells, which was then utilized to generate the ECL emission of polyluminol on the substrate [54]. A similar strategy was applied for ECL microscopy to image microRNAs (miRNAs) (Figure 4b) [55], whereby gold nanocages containing PMA and DNAs that can recognize miRNA-21 were utilized as a probe. Following the recognition of miRNA-21, PMA was released inside the cells and ROS were produced and visualized. In the above report, HeLa cells were cultured on a fluorine-doped tin oxide (FTO) conductive electrode and luminol was used for the ECL imaging. This probe can also be utilized for chemotherapy and photothermal therapy applications.

A capillary-based ECL device was also reported to visualize the H_2_O_2_ inside single cells (Figure 4c) [56]. In this case, the capillary was coated with poly(vinyl chloride)/nitrophenyl octyl ether (PVC/NPOE) and gold and a mixture of chitosan and luminol was used to fil the tip. Due to the small tip employed, a capillary was inserted inside the cell, and the ECL derived from the intracellular H_2_O_2_ was successfully observed.

Ji and co-workers reported the detection of GSH in a single Ramos cell through the coupling of capillary electrophoresis with ECL [22]. GSH is a common non-protein thiol class species present in living cells and plays an important role as an endogenous antioxidant. In their system, GSH acted as a coreactant of Ru(bpy)_3_^2+^. More specifically, a single Ramos cell was injected and lysed in a fused-silica capillary by electrophoresis. Upon the elution of GSH from the capillary, a luminescence peak was observed due to the reaction of GSH and Ru(bpy)_3_^3+^ in the detection cell.

### 4.2. ECL Detection of Biomolecules on Cells

**Figure 5:**
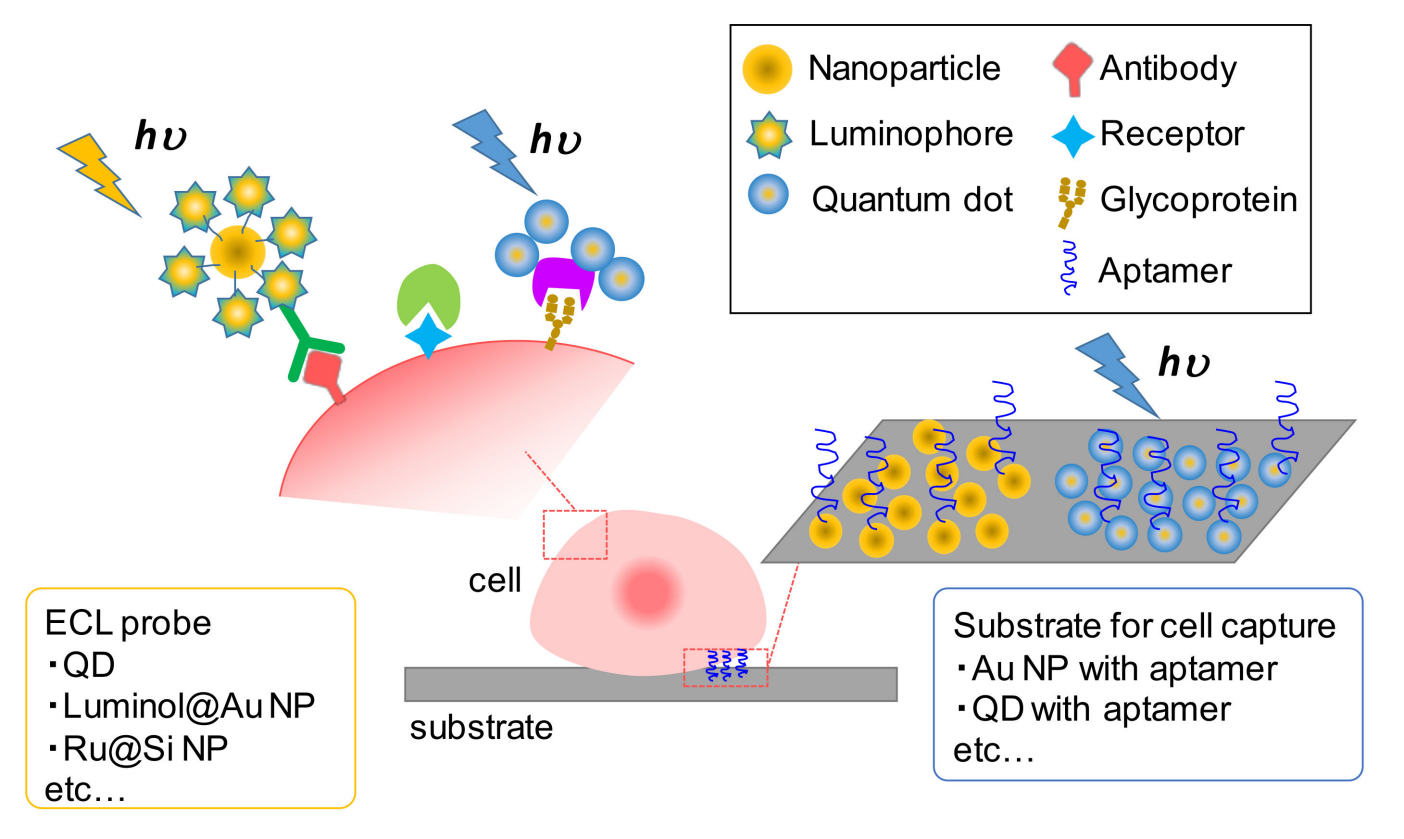
Conceptual image of cytosensing by the ECL technique. The choice of ECL probes and substrates for cell capture varies among different kinds of nanomaterials and their combinations.

The detection of specific cells and their populations is of key importance in the area of pathology, especially in the context of cancer diagnosis and prediction. For example, for the recognition of target cells, the antibodies, receptors, and surface glycans expressed on cells have been detected as indicators. With the development of nanotechnology, various nanomaterials, such as quantum dots (QDs) and silica- and metal-based nanoparticles, have been utilized to label these biomolecules due to the high volume-to-surface ratio that is favorable for their attachment. For ECL detection, QDs have been used as ECL emitters due to their unique optical/electrical properties, while silica- and metal-based nanoparticles have been utilized not only as carriers of ECL probes but also to enhance the luminescence. In this subsection, we introduce strategies of ECL detection based on the use of these materials, in addition to assemblies of ECL probes and substrates for cell capture (Figure 5).

The first studies of cancer cell detection utilizing QDs were reported by several groups in 2011. More specifically, Han and co-workers demonstrated the ECL detection of carbohydrate expression on living cells by utilizing the specific recognition of lectin to carbohydrate groups with the functionalization of the immobilized CdSe QDs [57]. In addition, Jie et al. reported an ECL cell-detection system whereby CdSe-ZnS-QDs was used as the signal probe [58]. They prepared dendrimer nanoclusters for binding a large number of CdSe-ZnS-QDs and target DNA, which improved the ECL signal. Around the same time, Chen’s group proposed the electrochemiluminescence resonance energy transfer (ERET) system, using CdS QDs as the ECL donor and energy transfer to Ru(bpy)_3_^2+^ as a receptor that emits luminescence at a different wavelength. Using this system, they demonstrated the quantitative detection of β2 microgloblin expressed on cells based on the energy transfer between the antibody-modified CdS QDs donor and the Ru(bpy)_3_^2+^ acceptor labelling the cell surface [27]. Subsequently, Yang’s group detected the EGFR expression level on MCF-7 cell surfaces using epidermal growth factor (EGF)-functionalized CdS QD-capped magnetic beads as the ECL probe [59].

Compared with traditional silicon-based QDs, carbon-based QDs are receiving growing attention due to their lower toxicity and environmentally friendly nature. For example, Liu et al. prepared graphene quantum dots (GQDs) coupled with surface villous Au nanocages (SVAu nanocages) as an ECL probe. These GQDs@SVAu nanocages were labeled with secondly antibodies and utilized in a sandwich-type cell assay to detect CA153 on the MCF-7 cell surfaces [60]. In addition, the Yang group fabricated ECL nanoprobes containing carbon QDs (CQDs) and demonstrated the single-cell detection of platelet adhesion to endothelial cells [61], in addition to evaluating the CD44 expression level of a breast cancer cell [62,63].

**Figure 6:**
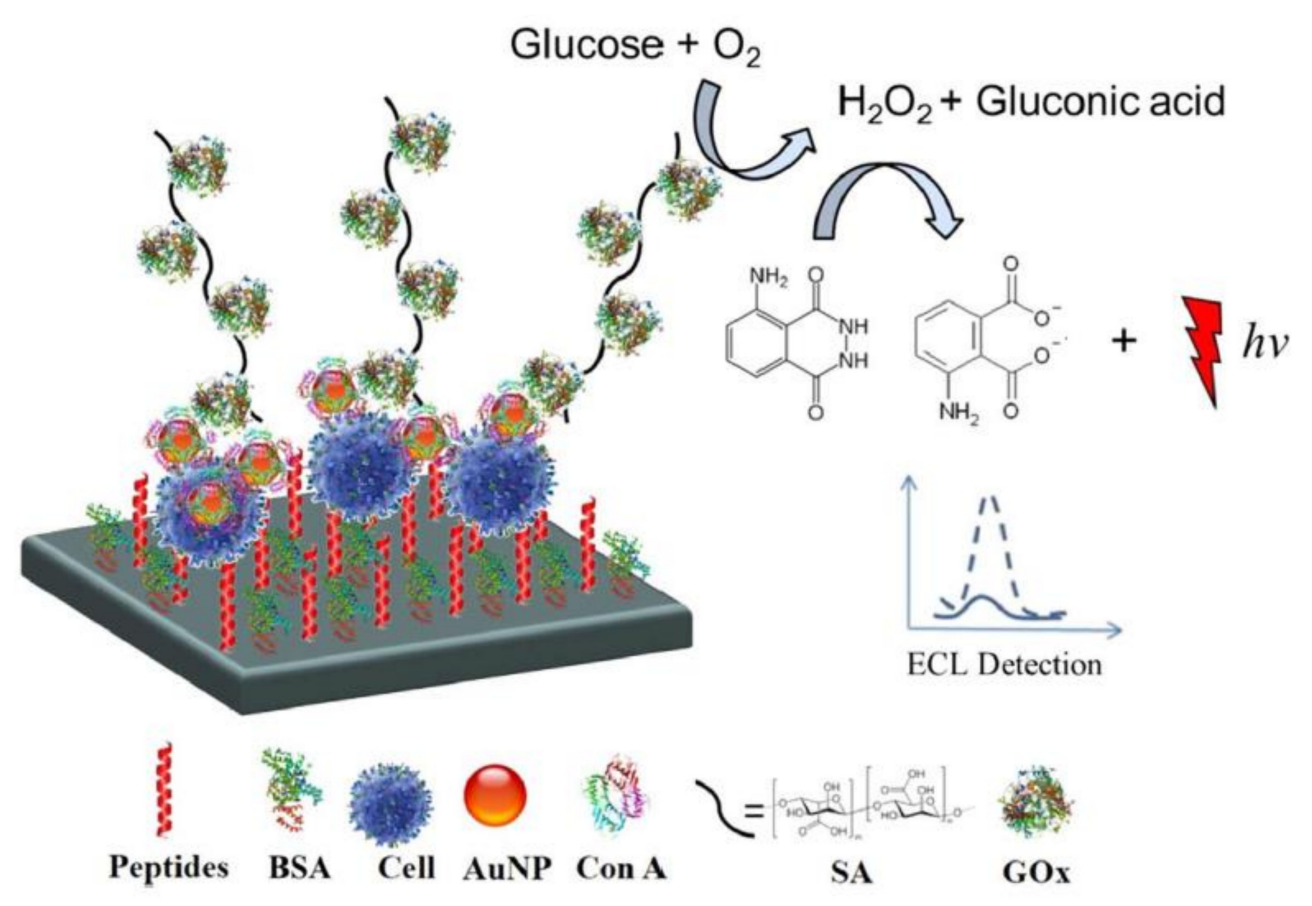
Schematic illustration of the luminol-H_2_O_2_ system with a glucose oxidase-conjugated sodium alginate (GOx@SA) nanoprobe for the detection of cell surface glycans. Glucose oxidase on the nanoprobe catalyzes the oxidation of glucose to produce hydrogen peroxide, which improves the ECL signal. Reproduced with permission from [67].

On the other hand, silica and gold nanoparticles (NPs) are favorably used as nanocarriers to conjugate larger amounts of luminophores or improve electron transfer from the electrode interface to the signal probes. For example, Ru(bpy)_3_^2+^ [64] and luminol [65] have been coupled with these nanomaterials for the design of efficient ECL signal systems. More specifically, Li’s group fabricated an ECL cytosensor based on concanavalin A (Con A)-integrating gold nanoparticle-modified Ru(bpy)_3_^2+^-doped silica nanoprobes (Au−RuSiO_2_ NPs). Silica NPs were used for doping large amounts of Ru(bpy)_3_^2+^, while Au NPs served not only as carriers of the proteins, but also electrocatalytic probes to improve the ECL signal. Taking the advantage of the specific binding of Con A to mannose, the in situ and dynamic evaluation of the cell surface *N*-glycan expression was demonstrated [26]. In another study, alkaline phosphatase (ALP) and Con A-coated Au NPs were employed to label cell surface glycans [66]. Due to the phenol products produced by ALP enzyme catalysis, the ECL signal of Ru(bpy)_3_^2+^-TPrA was significantly inhibited in accordance with the expression of surface glycans. Another approach was reported by Zhang and co-workers, whereby the luminol-H_2_O_2_ system was used to detect carbohydrate expression on a cell surface [67]. They prepared Con A-modified Au NPs as multiple-site combined probes and glucose oxidase-conjugated sodium alginate (GOx@SA) as the nanoprobe. In this system, Con A served to recognize the cell surface carbohydrates and capture the GOx@SA nanoprobes. The presence of glucose oxidase on the nanoprobes catalyzes the oxidation of glucose to produce H_2_O_2_, which significantly improves the ECL signal of luminol (Figure 6).

During investigations into the in situ detection of cellular functions, the toxic side effects of the coreactants must be taken into consideration. Thus, to address such effects, a self-enhanced ECL system was proposed in which the luminophores and the coreactant groups are covalently linked through organic synthesis. Since the electron transfer between the luminophore and the co-reactive group occurs intramolecularly, no additional coreactants are required to obtain the ECL signal. For example, Liang et al. synthesized self-enhanced ECL ruthenium−silica composite nanoparticles (Ru−N−SiNPs) labeled annexin V as signal probes. In this system, Ru(dcbpy)_3_^2+^ was oxidized to Ru(dcbpy)_3_^3+^, and the secondary amine (−NH−) in the intra-coreactant was oxidized to the radical cation (−N^•+^H−) both on the electrode surface. Subsequently, the excited form of Ru(dcbpy)_3_^2+^* was generated by the reaction with −N^•+^H− and Ru(dcbpy)_3_^3+^, resulting in significant ECL emission when Ru(dcbpy)_3_^2+^* was relaxed to the ground state. Since annexin V has a specific binding property with phosphatidylserine, which has been investigated as a biomarker for apoptotic cells, the proposed ECL system was employed for the detection of cell apoptosis, thereby demonstrating its potential for use in anticancer drug screening [68]. In addition, Cui et al. prepared a self-electrochemiluminous graphene oxide-capped Au@L012 nanocomposite as the label with the CEA antibody to detect the attomolar quantities of the CEA antigen [69]. Their strategy was to load as much L012 as possible to the Au NPs to maximize the luminescence intensity. Therefore, L012 molecules (i.e., the luminol analogue) were linked with poly(diallyldimethylammonium chloride) (PDDA) to form positively charged PDDA&L012 pairs, which were modified on the negative-charged Au@nafion nanoparticles to construct an Au@nafion@PDDA&L012 (Au@L012) complex. We would like to point out here that huge amounts of research have been dedicated to the use of nanomaterials as ECL probes [70,71], and so we would like to apologize for mentioning only a few here. For further studies, readers can refer to a number of excellent reviews of ECL nanoprobes [72].

### 4.3. Applied ECL Sensing Techniques

**Figure 7:**
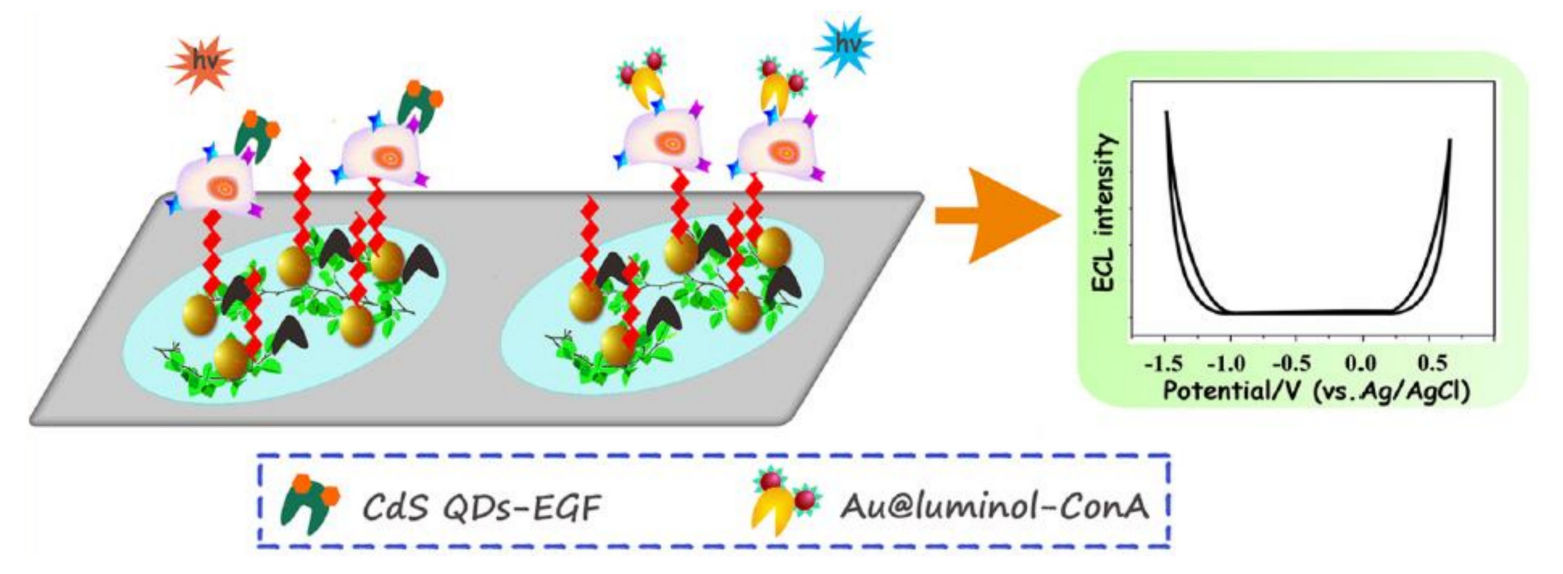
Schematic illustration of the dual ECL signal system. ECL signals at cathodic and anodic potentials can be obtained using CdS quantum dots (QDs) and luminol as ECL emitters, respectively. Reproduced with permission from [76].

Bimodal electrochemiluminescence systems allow the highly accurate and versatile analysis of cells [73,74,75]. For example, Zhou et al. demonstrated the multiplex detection of cell-surface receptors, mannose, and EGFR using a dual-ECL signal system. This system employed two kinds of ECL emitters, namely luminol-capped gold nanoparticles (Au@luminol) and CdS quantum dots (CdS QDs), which served as the labeling probes for mannose and EGFR, as well as the anodic and cathodic nanoemitters for the ECL signals (Figure 7). The two ECL probes were introduced to label the cells on two spatially resolved areas of a single sensing interface. During the potential sweep, well-separated and sensitive ECL signals at +0.5 V and −1.2 V were observed, demonstrating the sensitive and sequential detection of multiple receptors on the cell surface [76]. Recently, Ding et al. proposed a bimodal ECL system based on an internal standard method [77]. More specifically, CdTe QDs tagged with a cancer cell aptamer (CdTe-Apt 2) were used for detection, while luminol molecules were used as the internal standards. By comparing the differences in sensitivity between the double-peak ECL signals with those of the target analytes, this system allowed the quantification of cells in complex media. They also demonstrated the detection of target cells among cell suspensions containing different kinds of cells and also in human serum.

ECL cytosensors have also been combined with microfluidic and lab-on-a-chip techniques with the aim of achieving fast clinical diagnosis and point-of-care testing [78]. In this context, a paper-based ECL system was proposed by Yu and co-workers to give an easy-to-use and inexpensive ECL cytosensor [60]. More specifically, they fabricated Au nanoflowers on the surface of a paper working electrode and introduced AuPd nanoparticles as the labeling probe. Taking advantage of their large surface area, superior conductivity, and favorable catalytic activity toward H_2_O_2_ as a coreactant of luminol and the CdTe QDs group, which was used as the ECL emitter, alpha-fetoprotein (AFP) and the CEA antigen on the MCF-7 cell surface were detected by this system [79]. Furthermore, Lyu et al. utilized a mesoporous silica film (MSF) as a template to load Au NPs [80]. Owing to the ultra-small pore diameter of MSF, the Au NPs could be distributed uniformly without aggregation. The loading of Au NPs inside the MSF also contributes to preventing electrode fouling over long-term use. This Au NP-loaded MSF was prepared on ITO glass, and the H_2_O_2_ released from macrophage cells present on the film was detected. For on-chip cell analysis, the BPE system can also be considered a promising technique suitable for application in ECL devices. Since numerous studies have been published on ECL devices based on the BPE system, these will be discussed in the following section.

## 5. Bipolar Electrode (BPE) Devices for ECL-Based Cell Analysis

**Figure 8:**
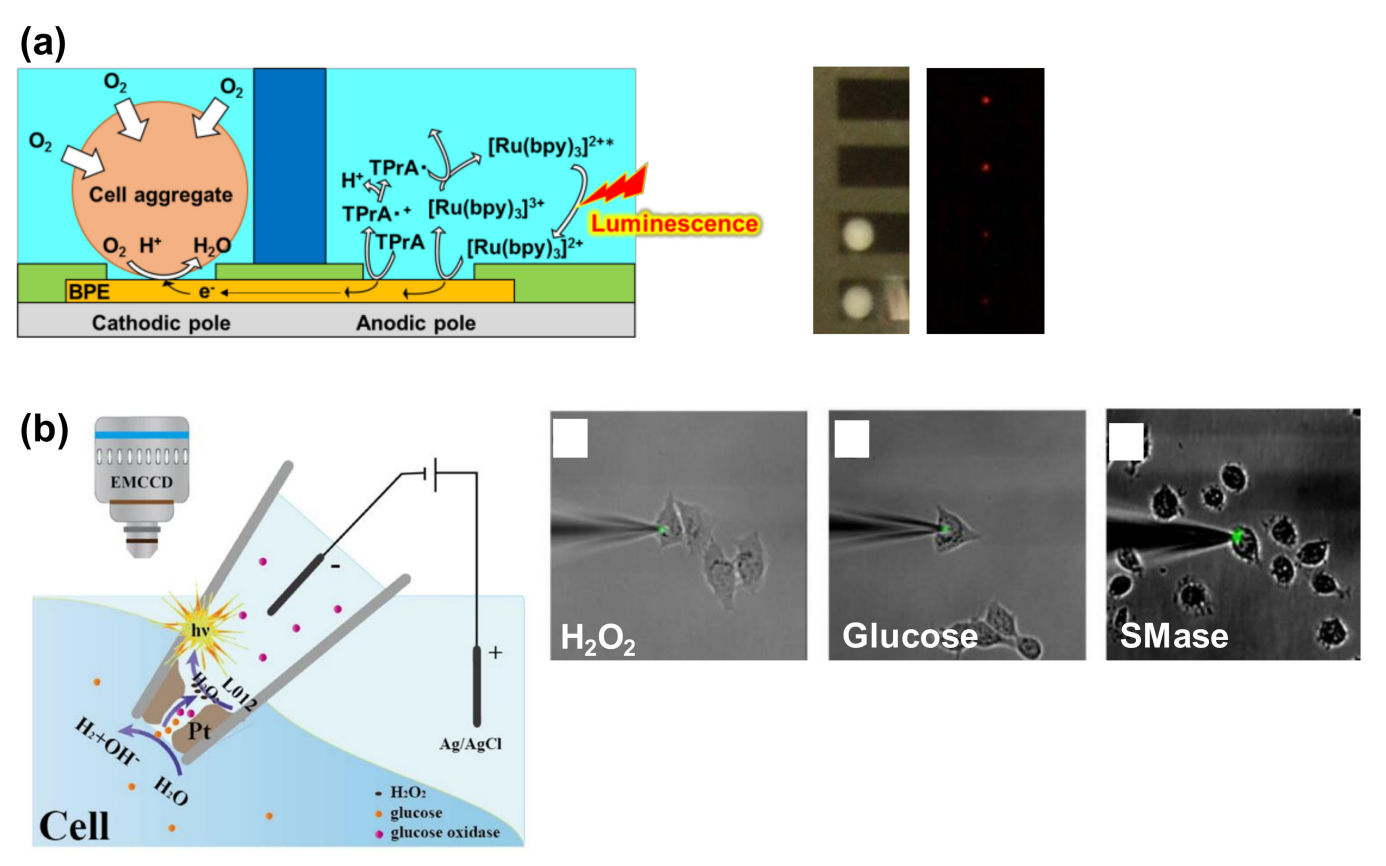
BPE devices for ECL-based cell analysis. (**a**) Detection of the respiration activity of the cell aggregates. A closed BPE system was used. Cell aggregates were set on cathodic poles, and ECL signals were acquired at the anodic poles. When the respiration activity was high, the ECL signal was low. Reproduced with permission from [90]. (**b**) Intracellular analysis using the nanopipette. The open BPE system was prepared in the nanopipette and inserted into a single cell. When the intracellular molecules diffused into the nanopipette, the ECL emission was observed. Overlaid images of ECL and bright field allow the visualization of H_2_O_2_, glucose, and sphingomyelinase (SMase) in a single cell. Reproduced with permission from [97].

BPE systems are widely utilized for ECL-based cell analysis (Figure 8), and these BPE–ECL systems can be roughly categorized into two types, namely open and closed BPE systems. In the open BPE system, anodic and cathodic poles are immersed in the same solution. In one literature example, over 1000 ECL sensors were prepared in a simple open BPE chip [81]. In contrast, in the closed BPE system, the solutions of the anodic and cathodic poles are physically separated. Although the structures of the closed systems are more complex than those of the open system, the culture medium can be separated from the ECL chemicals, which is useful for cell analysis. In addition, BPE–ECL devices can be categorized into chip and probe devices. Thus, in this section, we summarize open- and closed chip devices in addition to probe devices for ECL cell analysis.

### 5.1. Open BPE ECL Devices

Glycoproteins play essential roles in cell differentiation, cell proliferation, and cell-cell communications, and so their detection is of particular interest. In this context, open bipolar ECL techniques have been applied in the detection of cell surface glycoproteins [82], whereby dissolved oxygen was reduced on the cathodic pole and Ru(bpy)_3_^2+^ and TPrA were oxidized on the anodic pole to result in ECL emission. At the anodic pole, captured DNA is modified and a ferrocene (Fc)-labeled Mucin-1 aptamer that can hybridize the captured DNA and target cells is introduced onto the anodic pole. In this system, a U-shaped ITO was used, and the ECL signal was regulated by the electrochemical reactions of Fc due to the quenching effect of Fc^+^ on ECL emission. Using this strategy, the ECL intensity increased as the concentration of the target cells increased. Open bipolar ECL techniques were also applied for the analysis of folate receptors (FRs) [83]. In this case, folate acid (FA) was detected due to the inhibition effect of FA on the ECL of the Ru(bpy)_3_^2+^ and TPrA system. Since the quantity of FA was dependent on the FRs, the concentration of FA was measured, and the FA uptake behavior by the FRs on HL-60 cells was successfully analyzed.

### 5.2. Closed BPE ECL Devices

Closed BPE systems, in which both poles of the BPE are separated, enables the use of different solutions at the anode and cathode, and so can prevent the exposure of cells to the luminophore solution. Moreover, compared to open BPE systems, it is possible in the closed systems to enhance the electrode interface potential at both poles [84].

As an example, a U-shaped ITO bipolar device has been applied in the detection of cancer cells [84], whereby the ECL emission of Ru(bpy)_3_^2+^/TPrA was obtained at the anodic pole. At the cathodic pole, the antibodies were modified to capture target cancer cells, and a graphene oxide (GO)-Au/SH-aptamer was then introduced for the sandwich-type assay. Since the electroconductivity increased in the presence of target cells, the ECL at the anodic pole increased. Moreover, the sandwich-type assay strategy resulted in improvements in both the cell selectivity and the reliability of the ECL signal.

In addition, it has been reported that cellular respiration can be monitored using conventional electrochemical devices [85,86,87,88,89], and a closed bipolar ECL technique has also been applied for the detection of cell respiratory activities (Figure 8a) [90]. During detection, the dissolved oxygen was reduced at the cathodic poles, and TPrA and Ru(bpy)_3_^2+^ were oxidized at the anodic poles to generate ECL emission. Thus, the ECL signal was dependent on the concentration of dissolved oxygen. For cell analysis, the cell aggregates were set on the cathodic poles and the oxygen concentration close to the cells was reduced. The ECL signal was therefore low when the cell aggregates consumed the dissolved oxygen. In the described study, by taking the advantage of the BEP structure, 32 samples were evaluated simultaneously with only a single power supply.

A further study reported the combination of a closed bipolar ECL system with a paper-based device for the detection of intracellular H_2_O_2_ [91], whereby luminol/Au NPs were used as signal probes. A closed bipolar system has also been applied for counting cancer cells via the detection of cell extracts [92]. In this system, a dual-bipolar electrode array device containing three separated reservoirs filled with a buffer, TPrA-Ru(bpy)_3_^2+^, and luminol was employed. Upon the addition of DNAzyme and H_2_O_2_ derived from the extracts of cancer cells into these reservoirs, the orange ECL of Ru(bpy)_3_^2+^ was quenched and the blue ECL of luminol-H_2_O_2_ was turned on. Through this dual color ECL, the highly accurate detection of cells was demonstrated, thereby indicating that a visual color-switching ECL can provide cancer diagnosis for multiple assays. In another report describing the counting of cancer cells [93], the ECL emission of luminol-H_2_O_2_ was induced at an anodic pole, and the target cells were captured at a cathodic pole. When the target cells were adsorbed onto the BPE cathode via the appropriate antigen, the BPE resistance was slightly reduced. This decrease in resistance was reflected in the dissolution time of Ag modified on the anode surface and by measuring the time until ECL was generated; since the dissolved Ag suppresses the ECL generation of luminol, the count of cells on the cathode could be measured. Owning to the slight difference in conductivity, low numbers of cells could be successfully detected.

Although a cell analysis has yet to be demonstrated, both our group and the Zhang group have reported closed BPE arrays containing multiple sensing points [94,95,96]. One such system was fabricated using pyrolyzed carbon, and more than 100,000 BPEs were packed into an area of 1 cm^2^, thereby resulting in a particularly high resolution of the electrochemical images. Since the ECL reaction and the electrochemical reactions of biosamples can be separated using the closed bipolar system, these BPE arrays will likely be applicable for use in cell analysis.

### 5.3. BPE Probe Devices

In the context of BPE probe devices, nanopipettes have been applied in bipolar ECL for intracellular wireless analysis (Figure 8b) [97]. Inside the tip, Pt was deposited as an open bipolar electrode and the porous structure of the Pt deposit allowed intracellular molecules to be transported into the nanopipettes. The intracellular molecules then came into contact with the ECL reagent (L012) and the enzymes (or enzymes and substrates) filling the nanopipettes; ECL emission was produced at the open BPE due to the production of H_2_O_2_ followed by the reaction of the enzymes and target molecules. The study demonstrated the ECL detection of intracellular H_2_O_2_ from cells stimulated with PMA, in addition to glucose detection using glucose oxidase and the detection of sphingomyelin using sphingomyelinase (SMase), alkaline phosphatase, and choline oxidase (Figure 8b). The ECL detection of sphingomyelin with these enzymes is described in another report [98].

The use of scanning bipolar electrochemical microscopy has also been reported [99], although cell analysis has yet to be demonstrated. Since the probe present in the microscope contained multiple electrodes, it could be used to map the release of analyte species in real time without the requirement for a time-consuming scanning process. This group also reported O_2_ detection using the same system [100] and considered that this system could be utilized for cell analysis.

## 6. Conclusions and Perspectives

This review introduced various approaches for the analysis of mammalian cells based on the use of electrochemiluminescence (ECL) methods. The different types of ECL and ECL systems for cell analysis are summarized in Table 1 and Table 2, while the various target analytes are summarized in Table 3. In terms of representative luminophores, we focused on Ru(bpy)_3_^2+^ and luminol-based systems; based on or derived from their luminescence mechanisms, various imaging techniques and sensing strategies for single cell or ultratrace amounts of cell-derived molecules have been developed. Indeed, the described reports demonstrate that ECL is a promising technique for cellular analysis; however, a number of issues remain in terms of widening the application of ECL. For example, the cytotoxicities of Ru(bpy)_3_^2+^ and tri-*n*-propylamine cannot be ignored in the context of imaging living cells or in vivo imaging. In addition, a lowering of the driving potential would be desirable to prevent the degradation of the electrodes and electrical damage to cells. Thus, the investigation of novel ECL luminophores and coreactants remains an ongoing challenge. In terms of diagnosis, reusable, low-cost, and simplified devices exhibiting the high resolution and sensitivity of ECL are favorable. In this context, bipolar electrode (BPE) systems can be considered promising for the simplification of the electrochemical apparatus. Taking advantage of the wireless system, the development of multiplex bioassays using the BPE system is currently a topic of interest. Overall, we could conclude from the selection of studies discussed herein that the ECL technique is highly versatile and will soon become an essential analytical platform for mammalian cells.

## Figures and Tables

**Table 1:** Device types for electrochemiluminescence (ECL)-based cell analysis.

Device Types	References	Notes
Chip devices not for microscopic imaging	[74,78,79,80,91]	NA
BPE devices	[82,83,84,90,91,92,93,97]	NA
Probe devices	[56,97]	Intracellular detection
ECL microscopes for single-cell imaging	[21,23,39,40,41,42,43,44,45,49,50,52,53,55]	NA

**Table 2:** Target analytes.

Target Analytes	References	Notes
Cellular membrane	[39,40]	NA
Cell adhesion	[21]	NA
Cellular H_2_O_2_	[33,43,44,45,50,56,80,91,97]	NA
Glutathione	[22]	NA
Cholesterol	[43,46,47,48,49]	NA
Intracellular glucose	[52,53,97].	NA
SMase	[97,98]	NA
*N*-glycan on cell surface	[66,75]	NA
Mucin 1 on cell surface	[34,76,82]	NA
Mannan on cell membrane	[57]	NA
CEA on cell membrane	[41,69,74,79]	NA
AFP on cell membrane	[74,79]	NA
PSA on LNCaP cell	[24]	BPE device
CA153 on breast cancer cell	[60]	NA
CD44 on breast cancer cell	[62,63]	NA
β2 Microglobulin	[27]	NA
E-selectin on endothelial cell	[61]	Platelet adhesion
Phosphatidylserine	[68]	Cell apoptosis
EGFR	[39,59,76]	NA
FA and FR	[83]	BPE device
Intracellular telomerase	[54,73]	NA
miRNA	[55]	NA
mRNA	[23]	NA
Cancer cells	[25,58,65,67,77,78,82,84,91,92,93]	NA
Respiration activity	[90]	BPE device
Apoptosis	[42]	Detection of EGFR and PS

**Table 3:** Types of ECL systems.

ECL Systems	References	Notes
Ru(bpy)_3_^2+^-TPrA	[24,26,39,40,66,82,83,84,90]	NA
Ru(bpy)_2_(dcbpy)-TPrA	[25]	NA
Ru(bpy)_3_^2+^-HEPES	[21]	NA
Ru(bpy)_3_^2+^-GSH	[22]	NA
Ru(dcbpy)_3_^2+^-silica-NPs	[68]	Self-enhanced ECL with intracoreactant
Luminol-H_2_O_2_	[46,47,55,56,65,67,80,91,93,98]	NA
L012-H_2_O_2_	[42,43,44,45,48,49,50,52,53,97]	NA
Polyluminol-H_2_O_2_	[54]	NA
L012	[41,69]	Square wave voltage with high frequency [41] Graphene oxide-capped Au NPs [69]
ABEI-H_2_O_2_	[33]	NA
ABEI functionalized MOFs	[34]	O_2_^•−^ as a coreactant
g-C_3_N_4_-S_2_O_8_^2−^	[42]	NA
Quantum dots	[23,57,58,59,60,61,62,63,78]	NA
Ru(bpy)_3_^2+^-S_2_O_8_^2−^/CdS QDs	[27]	Resonance energy transfer
Luminol-H_2_O_2_/CdS NCs	[73,76]	Dual ECL signal system
Luminol/Ru(bpy)_3_^2+^-S_2_O_8_^2−^	[74]	Dual ECL signal system
CNNS/Luminol-H_2_O_2_	[75]	Dual ECL signal system
Luminol/CdTe-S_2_O_8_^2−^	[77]	Dual ECL signal system
Luminol/CdTe-H_2_O_2_	[79]	Dual ECL signal system
Luminol-H_2_O_2_/Ru(bpy)_3_^2+^-TPrA	[92]	Dual ECL signal system with BPE device
